# Bioactive Compounds of the Mediterranean Diet as Nutritional Support to Fight Neurodegenerative Disease

**DOI:** 10.3390/ijms24087318

**Published:** 2023-04-15

**Authors:** Gianluca Antonio Franco, Livia Interdonato, Marika Cordaro, Salvatore Cuzzocrea, Rosanna Di Paola

**Affiliations:** 1 Department of Chemical, Biological, Pharmaceutical and Environmental Sciences, University of Messina, 98125 Messina, Italy; 2 Department of Biomedical, Dental and Morphological and Functional Imaging, University of Messina, Via Consolare Valeria, 98125 Messina, Italy; 3 Department of Veterinary Sciences, University of Messina, 98168 Messina, Italy

**Keywords:** natural substance, neurodegenerative diseases, oxidative stress, inflammation, diet

## Abstract

Neurodegenerative disorders are a widespread cause of morbidity and mortality worldwide, characterized by neuroinflammation, oxidative stress, and neuronal depletion. They include selective malfunction and progressive loss of neurons, glial cells, and neural networks in the brain and spinal cord. There is an urgent need to develop new and more effective therapeutic strategies to combat these devastating diseases because, today, there is no treatment that can cure degenerative diseases; however, we have many symptomatic treatments. Current nutritional approaches are beginning to reflect a fundamental change in our understanding of health. The Mediterranean diet may have a protective effect on the neurodegenerative process because it is rich in antioxidants, fiber, and omega-3 polyunsaturated fatty acids. Increasing knowledge regarding the impact of diet on regulation at the genetic and molecular levels is changing the way we consider the role of nutrition, resulting in new dietary strategies. Natural products, thanks to their bioactive compounds, have recently undergone extensive exploration and study for their therapeutic potential for a variety of diseases. Targeting simultaneous multiple mechanisms of action and a neuroprotection approach with the diet could prevent cell death and restore function to damaged neurons. For these reasons, this review will be focused on the therapeutic potential of natural products and the associations between the Mediterranean-style diet (MD), neurodegenerative diseases, and markers and mechanisms of neurodegeneration.

## 1. Introduction

The increasing incidence of neurodegenerative diseases (NDDs) and lack of cures or efficacious treatments has stressed the need to develop new treatments [1]. The most significant risk factor for the emergence of neurodegenerative disease is age, and the majority of neurodegenerative disorders are often present in the elderly [2]. Neurodegenerative diseases are characterized by progressive movement disorders, such as in Parkinson’s disease (PD), or memory loss, cognitive decline, and behavioral disturbances, such as in Alzheimer’s disease (AD) [3]. Despite the distinctions in etiology between the two diseases, they are both classic neurodegenerative diseases marked by the chronic, progressive loss of neurons and their synaptic connections, which manifests as a steady deterioration in functional ability [4]. The activation of microglia results in elevated production of cytokines and pro-inflammatory mediators and increased neuronal cell death, which are the hallmarks of neuroinflammation; along with oxidative stress, these are the major causes of the onset of these incurable neurodegenerative diseases [5]. There are currently few effective preventive or therapeutic measures against neurodegenerative diseases. Additionally, longer life expectancies have led to a rise in the prevalence of neurological disorders such as PD and AD [6,7,8]. Studies proving the protective and preventive effects of specific substances naturally found in food, known as nutraceuticals, on general health and well-being have grown over the past 20 years [9,10,11]. The significance of bioactive substances in the prevention of neurodegenerative diseases, which are on the rise nowadays, has recently been the subject of extensive investigation. Plants and plant parts are used for their fragrance, flavor, or therapeutic properties. There are several advantages associated with using plants and plant phytoconstituents as opposed to pharmaceutical products. Plant extracts and phytoconstituents have been shown to have biological properties, such as antidiabetic [12], antihyperlipidemic [13], free radical scavenging [14], and anti-inflammatory properties [15,16]. It has been established that dietary patterns (DPs) that increase the risk of metabolic and cardiovascular disorders also significantly increase the risk of dementia [17]. Proposed underlying mechanisms include: (l) data-driven approach utilizing factor or cluster analysis to create dietary patterns, (ll) hypothesis-driven approach using diet quality indices or scores (e.g., the Mediterranean model), and (lll) reduced-rank regression integrating elements of the first two approaches. DPs with higher intakes of fruits, vegetables, fish, nuts, and legumes and lower intakes of meats, high-fat dairy items, and sweets seem to be associated with lower odds of cognitive impairment or reduced risk of AD and PD, despite the disparities between the approaches [18]. Scientists have been trying to find effective treatments for neurodegenerative diseases utilizing natural compounds with plant origins more frequently in recent years. In consideration of all the studies that have been carried out recently [19,20,21] on bioactive compounds and the effects they have on inflammatory and oxidative diseases, this review aims to gather the latest recent discoveries to give the reader an overview on the potential benefits of bioactive compounds in the Mediterranean diet, their origin, and the molecular pathways on which they act in neurodegenerative diseases.

## 2. Mediterranean Diet

**Figure 1:**
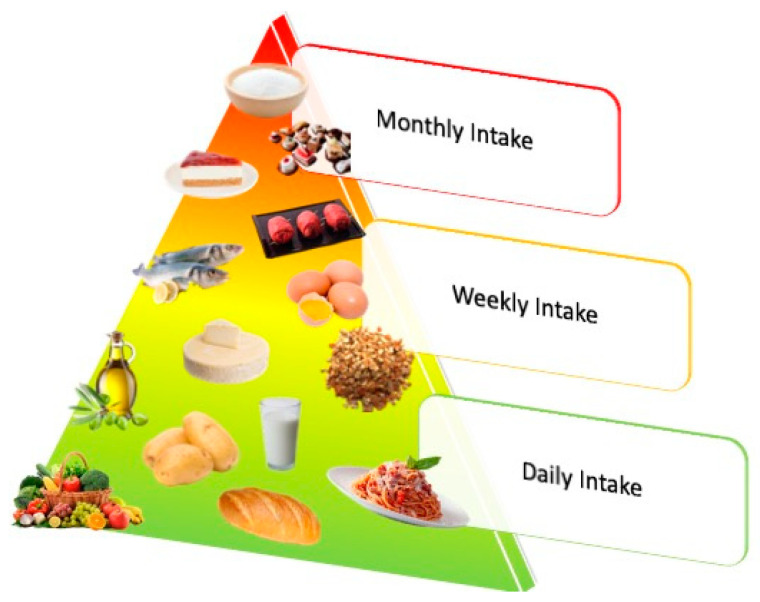
The food pyramid illustrates how to maintain a complete and balanced diet.

The expression “Mediterranean diet” (MD) is frequently used to describe the eating habits of those who reside around the Mediterranean Sea coast, predominantly in Greece, southern Italy, and southern Europe [22]. Diet, including wine if used in moderation, is a multisecular key component of health and well-being in the Mediterranean civilization. Mediterranean cuisine is characterized in an alimentation rich in fruits, vegetables, olive oil, fish, and infusions, which in turn are very rich in fiber, vitamins, polyunsaturated fatty acids, and oligoelements (Figure 1). In addition, wine, particularly red wine, has additional specific polyphenols with antioxidant properties, including resveratrol, procyanidins, and monophenols [23]. Consumption of fruits and vegetables such as oranges, pomegranates, berries, figs, and grapes is a source of dietary fiber, potassium, vitamin C, polyphenols (mostly flavones), and terpenes [24]. On the other hand, the Western diet, which is low in fiber and fruits, may be associated with AD or PD [17,25,26,27]. In contrast to the Western diet, the MD has a lot of fiber. The benefits of sticking to the MD may be mediated by the composition of the gut microbial community because fiber is a primary energy substrate for gut flora [28]. Different studies have been demonstrated that long-term consumption of an MD has positive benefits on a number of chronic diseases, including cancer, diabetes, and heart disease, in addition to protecting the brain against acute and chronic neurological disorders. These health advantages are related to the activity of flavonoids such as polyphenols and anthocyanins as well as the presence of antioxidants such as tocopherol, ascorbic acid, and carotene [29,30,31]. Below, we have highlighted the main foods present in the MD, useful in the treatment of neurodegenerative pathologies such as AD and PD. A Mediterranean diet may have a protective effect on the neurodegenerative process because it is rich in antioxidants, fiber, and omega-3 polyunsaturated fatty acids (Table 1). Several nutrients have been shown to have positive effects on the way neurodegenerative diseases develop. These include caffeine, some probiotic bacteria, polyphenols, curcumin, vitamins B6, B12, folic acid, unsaturated fatty acids, lecithin, and glutathione. In contrast, the development of these diseases is accelerated by a diet rich in saturated fatty acids and branched-chain amino acids (BCAAs) [32]. The first clinical research on this issue was undertaken as part of the WHICAP study (Washington Heights-Inwood Columbia Aging Project) in New York. Numerous articles from this initiative have shown that foods from the Mediterranean diet are beneficial for avoiding brain disorders and protecting the brain from aging and the development of degenerative diseases such as AD [33]. The first interesting study to hypothesize this association was the one by Scarmeas et al. in 2006 [34]. The authors studied a group of 2258 people whose cognitive performance was assessed every 1.5 years for a total of four years. By the end of the study, 262 instances of Alzheimer’s disease had been discovered. A higher adherence to the Mediterranean diet reduced the chance of acquiring Alzheimer’s disease by approximately 10% in this cohort (RR: 0.91, 95% CI 0.83–0.98) [34]. The Mediterranean diet was found to be helpful not only in terms of Alzheimer’s disease incidence but also in terms of cognitive decline, as indicated by a decrease in the score of a cognitive function test such as the Mini-Mental State Examination test. An investigation by WHICAP of the same project, conducted on a cohort of 1393 individuals with normal cognitive function at baseline, found that adherence to the Mediterranean diet was associated with a lower risk of cognitive deterioration after 4.5 years of follow-up (HR: 0.52, 95% CI 0.30–0.91) [35].

### Mediterranean Diet and Neurodegenerative Diseases

Components of the Mediterranean diet and their effects on cognitive performance in Parkinson’s and Alzheimer’s disease are listed in the table below (Table 2).

It should be noted that these findings are based on observational studies and that further research is needed to confirm these effects on cognition in people with Parkinson’s and Alzheimer’s disease. Furthermore, the Mediterranean diet may be only one aspect of a broader approach to promoting brain health, such as regular exercise, adequate sleep, and stress reduction.

## 3. Alzheimer’s Disease

Alzheimer’s disease is the cause of one of the most common types of dementia. The incidence of Alzheimer doubles every 5 years after the age of 60, increasing from a prevalence of 1% among those 60 to 64 years old to up to 40% of those aged 85 years and older [55]. AD is an aging brain condition characterized by the production of two major protein aggregates: senile plaques and neurofibrillary tangles, both of which are implicated in the process leading to progressive neuronal degradation and death [56].

Memory loss, agitation, and apathy are all related with degeneration in cholinergic neuron-rich regions, including the nucleus basalis of Meynert, frontal cortex, and the anterior and posterior cingulate cortex [57].

Age is the main risk factor for AD development; however, old age alone does not cause this illness. Additional risk factors include having one or more apolipoprotein gene E4 alleles (APOE4), having a family history of AD, having inadequate occupational and educational skills, having cardiovascular risk factors, and having experienced moderate to severe brain injuries [57]. According to the cholinergic hypothesis, the gradual loss of limbic and neocortical cholinergic innervation in AD is responsible for the decrease in memory, learning, attention, and other higher brain processes [58]. Furthermore, neurofibrillary degeneration in the basal forebrain is most likely the major source of cholinergic cell malfunction and mortality in this area, resulting in broad presynaptic cholinergic denervation. The acetylcholinesterase inhibitors (AChEIs) improve the availability of acetylcholine at synapses and have been shown clinically to slow cognitive deterioration in AD [59]. Other neurotransmitters that might be involved in the disease include dopamine, serotonin, glutamate, and GABA. However, the specific role of these neurotransmitters in Alzheimer’s pathology is not yet fully understood, and further research is needed to investigate this question [60].

### 3.1. AD Stages

AD is characterized by several different stages; the first is represented by the Preclinical stage. Subjective cognitive decline (SCD), a situation in which self-perception begins to deteriorate before the emergence of actual cognitive impairment, is thought to represent an early clinical symptom of AD. SCD has been prospectively linked to underlying AD pathology, and it is thought to likely accelerate memory decline [61].

These neuropathological alterations begin in the entorhinal cortex and hippocampal formations and progress to various temporal, parietal, and lastly frontal association cortices; from a symptomatologic point of view, subjects at this stage present mild memory loss with relative sparing of long-term memories and no significant impairment in their daily activities [62]. The second stage is represented by the Moderate form of AD.

In this phase, cognitive symptoms start manifesting; pathological damage progresses to the parts of the brain responsible for language, thinking, and sensory processing (cerebral cortex). Aside from a rise in the intensity of symptoms of the preceding stages, behavioral issues and social retreat tendencies emerge. This is followed by a linguistic dysfunction and a loss of visuospatial abilities. It is worth noting that subjects at this time have difficulty identifying their loved ones [63].

Individuals suffering from delusions may belong to a diverse group: The most prevalent late-life delusions, such as delusions of stealing, occur earlier in the AD course than misidentification delusions, such as TV sign and phantom boarder, which are linked to advanced dementia and higher cognitive impairment [64,65]. Subjects entirely lose their independence for daily tasks at this phase. The pathological damage at this stage is thought to encompass the whole brain. Systemic symptoms such as difficulties executing learned motor tasks (dyspraxia), olfactory dysfunction, and sleep abnormalities, along with extrapyramidal motor indications such as dystonia, akathisia, and parkinsonian symptoms begin to develop [63].

### 3.2. AD Biomarkers

Physiological, biochemical, or anatomical changes that may be tested in vivo and certain pathological alterations that can be identified in a disease are known as “biomarkers”.

The most well-known biomarkers related to AD are the increase in the levels of beta-amyloid peptide (Aβ), total tau (t-tau), and phospho-tau (p-tau) in cerebrospinal fluid (CSF). Multiple hypotheses have been suggested regarding the pathology of AD, such as the tau hypothesis in which β-amyloid plaques are involved [66]. The neuroprotective function of reactive astrocytes is mediated by regulating the Amyloid ß-linked (Aβ-linked) neural injury, degradation, and Aβ metabolism, hence, generating a protective barrier against Aβ deposits [67]. According to the amyloid hypothesis, the buildup of Aβ plaques acts as a powerful catalyst for the formation of neurosis and tangles—two processes that result in the neurodegeneration and apoptosis seen in AD. Plaques and NFTs are produced through several mechanisms related to Aβ over-generation/deposition and tau protein hyperphosphorylation/deposition, respectively [68]. Aβ peptides contain 39–43 amino acid residue proteins enzymatically cleaved by the action of β-secretase and Γ-secretase on a protein called amyloid precursor protein (APP) [69]. When utilized as a tracer in positron emission tomography (PiB-PET), the Pittsburgh compound B (PiB), a particular ligand of Aβ, enables the analysis of cerebral Aβ load and Aβ spatial distribution in vivo. [70]. PET imaging with a 2-deoxy-2 [18F] fluoro-D-glucose tracer (FDG-PET) analyzes glucose metabolism in the brain and is used to assess neuronal and glial function. The FDG-PET signal diminishes in AD, which is compatible with glucose hypometabolism and synaptic dysfunction, and it also exhibits a distinct topographic distribution pattern [71].

Unusually elevated levels of Aβ have been proposed by Jack and colleagues as the first event leading to the production of cerebral amyloid plaques. This would be reflected in lower levels of Aβ in CSF and a higher amyloid burden in PiB-PET, and these changes would occur while the patients were still cognitively normal. Following that, there would be an increase in CSF tau abnormalities, followed by changes in FDG-PET. These are neuronal dysfunction and neurodegenerative biomarkers that correspond with the severity of clinical symptoms. Current consensus declarations have stressed the need of early detection; hence, sensitive indicators that may act as adjuncts to current clinical and neuropsychological testing to assist diagnosis and/or monitoring of early brain alterations indicative of AD are urgently needed. Such indicators may also aid in early intervention studies aimed at preventing or slowing disease development. Traditional structural neuroimaging, such as computed tomography (CT) or magnetic resonance imaging (MRI), has long been used to aid in the identification of memory impairments and is suggested for routine AD evaluation [72].

## 4. Parkinson’s Disease

After Alzheimer’s disease, PD is the second most prevalent neurodegenerative condition, affecting 1% of people over 60 and up to 4% of people over 80 [73,74]. PD is a progressive neurodegenerative condition defined pathologically by dopaminergic neuron loss in the substantia nigra and the development of protein inclusions known as Lewy bodies. Previously, the condition was thought to be primarily a movement disorder, with a tetrad of motor impairments such as resting tremor, bradykinesia, postural instability, and stiffness of the neck, trunk, and limbs [75]. The primary motor characteristics of the condition are caused by the ensuing dopamine deficit, which is the focus of current symptomatic treatments. The buildup of intracytoplasmic inclusions containing α-synuclein is linked to pathological alterations in the substantia nigra. Aggregates of α-synuclein have also been discovered in numerous different peripheral and central areas of the nervous system [76,77]. Inherited Parkinson’s disease, to be distinguished from idiopathic Parkinson’s disease with unknown etiology, is influenced by both hereditary and environmental factors, while its exact etiology is known. The primary source of dopaminergic projections to the basal ganglia, the substantia nigra pars compacta (SNpc), is where neuronal damage and death in PD occur. Development of disease-modifying medications is centered on a number of cellular pathways, including mitochondrial failure, oxidative stress, neuroinflammation, and improper protein degradation, which have all been linked to the etiology of PD [78]. The deficiency in the dopamine neurotransmitter in the SNpc causes disruption of the circuitry that governs movement and posture, resulting in symptoms such as slowness of movement and relaxation trembling. The end result of PD’s degenerative development is oxidative stress as a result of cellular dysfunction [79].

### 4.1. PD Stages

PD is characterized by the presence of five different stages, each of which has characteristic symptoms. However, symptoms vary and may worsen as the disease progresses.

The early stage of Parkinson’s is the mildest. Symptoms may be present but are not severe enough to impair daily activities or one’s lifestyle. At this point, symptoms are so mild that they are often overlooked. However, family and friends may notice alterations in gait, posture, or facial expressions. Tremors and other movement problems are usually limited to one side of the body, a unique feature of stage one PD [80]

Stage two PD has a moderate form, and symptoms are significantly more pronounced than those in stage one. Changes in facial expressions and rigidity may be more prominent, as well as tremors and shaking. Stage two is characterized by muscle rigidity that does not affect balance but delays completion of tasks. The person’s posture may begin to change, and difficulty walking may occur. At this stage, people sometimes have difficulty speaking and suffer from symptoms on both sides of the body (although one side may be only moderately affected). Although some chores may take longer for most people with stage two Parkinson’s, they can still live independently. Moving from stage one to stage two may take months or even years.

The middle stage of PD, stage three, represents a significant turning point in the course of the disease. The symptoms are in many ways like those of stage two. However, the chances of losing balance and slowing down are higher. In general, movements slow down. That is why falls increase in stage three. At this stage, PD has a major impact on daily activities, but people can still complete them [81]. Stage three and stage four Parkinson’s patients are distinguished by their independence. Stage four allows people to stand independently. However, movement may require the use of a walker or other type of aid. The severe slowing of mobility and reaction time in this stage of Parkinson’s prevents many people from living alone. Living alone at stage four or beyond can make it difficult or risky to perform many daily activities. The most advanced stage of PD is the fifth. Advanced leg stiffness can also cause standing to become blocked, making it impossible to move or stand. People in this stage often need wheelchairs and cannot stand without falling. To prevent falls, constant help is needed [82] In stages four and five, disorientation, hallucinations, and delusions can affect up to 50 percent of people [83].

### 4.2. PD Biomarkers

A biomarker identifies the specific disease state of an organism and assesses the course of the disease and the effectiveness of treatment. It could be a parameter that is physical, chemical, or biological [84].

Although no one biomarker has been suggested for the diagnosis of PD, they can be combined logically to forecast the disease’s status and course. Since determining the diagnostic criteria for a disease is vital for identifying and confirming the biomarkers, the problems in diagnosing PD make the search for biomarkers challenging [85]. Biochemical biomarkers can be investigated either in the CSF or in the blood [86,87].

The biofluid-based biomarker Glial Fibrillary Acidic Protein (GFAP) and its breakdown products (GFAP-BDPs) may be useful for diagnosing neurological diseases such as PD [85]. Brain-derived neurotrophic factor (BDNF) is a powerful inhibitor of neurotoxin-induced degeneration of dopaminergic neurons as well as apoptosis-mediated cell death. In order to enhance cognitive functioning in PD, neuroprotective medicines eventually incorporate the BDNF [88,89,90]. The decline of dopaminergic neurons is linked to the decreasing expression of BDNF in the substantia nigra, which is involved in controlling neuronal survival. Neuromelanin-containing cells in the substantia nigra are impacted by PD. Neurons that have accumulated neuromelanin appear to be a protective phenomenon that inhibits a variety of neurotoxic processes. In PD, dead neurons emit neuromelanin, which sets off a vicious cycle of neuroinflammation and, eventually, neuronal death [85,91,92].

### 4.3. Inflammation and Oxidative Stress in Neurodegenerative Diseases

In acute neurodegeneration, such as that following a stroke or brain injury, as well as in chronic neurodegenerative illnesses such as AD and PD, the microglia play a key role in the inflammatory response. Microglia are referred to as being “active” in chronic neurodegenerative diseases because they have changed their shape from that of the resting state and have elevated or expressed de novo a variety of cell surface or cytoplasmic antigens. Nearly all neuronal homeostasis disturbances are accompanied by the activation of microglia; in fact, changes at the synapse, along the axon, or at the neuronal cell body can all trigger microglia activation [93].

Studies on AD contain the most comprehensive descriptions of chronic inflammation linked to chronic neurodegeneration [94,95]. In fact, the end-stage pathology shows that there are numerous activated microglia in the areas of the brain where there is neuronal death and amyloid deposits. It has been demonstrated that these activated microglia exhibit higher quantities of MHC class II, integrins, and several cytokines, including IL-1, IL-6, and TNF. It has been demonstrated that the presence of these pro-inflammatory cytokines in acute brain injury may increase the size of the lesion and neuronal loss by having an adverse effect on already damaged neurons [94,95].

Therefore, it stands to reason that in AD, the presence of these cytokines could worsen the death of neurons already weakened by Aβ or by disruption of their cytoskeleton and axonal transport caused by tau accumulations. Other inflammatory mediators or ongoing inflammatory processes, such as the deposition of complement components, have also been identified in addition to the presence of these inflammatory cytokines. Intense research is being done to determine if this inflammatory response, albeit being highly unusual, contributes to the development of the disease or is only a byproduct of tissue aging [94]. The biological oxidants that cause oxidative damage are assumed to be the byproducts of endogenous and external activities involving oxygen and nitrogen. Reactive oxygen species are created during aerobic respiration, cellular metabolism, and pathogen defense [96]. The oxygen molecule’s chemical potential is determined by its electron structure (two unpaired electrons in its basic triplet state). It stimulates single-electron reactions (reduction of oxygen molecules in four single-electron processes), microsomal electron transport chains (ETC) (through cytochrome P-450 (CYP 450)), and oxidative burst activity in macrophages [97]. The high kinetics of chemical processes attained in elementary single-electron reactions are desired and are the source of reactive molecules that are either unwanted byproducts (respiration and metabolism) or exceed the specified criteria (defense process). Reactive oxygen species (ROS) and reactive nitrogen species (RNS) are the names given to these reactive molecules. Singlet oxygen (1O2), superoxide anion radicals (O2•), hydroxyl radicals (HO•), hydrogen peroxide (H2O2), nitric oxide (NO), and peroxynitrite anions (ONOO) are the most well-known reactive molecules [98]. It has been proposed that oxidative imbalance and the resulting neuronal damage play a vital role in the onset and development of AD. The excessive buildup of ROS in AD patients may cause mitochondrial dysfunction; however, the source of increased ROS generation and the precise processes driving the disturbance of redox equilibrium remain unknown [99]. Even in the early stages of PD, changes in antioxidant molecules have been identified. Glutathione (GSH), a tripeptide synthesized from glutamate, cysteine, and glycine, exerts a protective function on cell survival against oxidative stress [100]. GSH has been found to be decreased in the SNc of PD patients; albeit, this discovery is not exclusive to PD [101].

## 5. Bioactive Compounds

**Figure 2:**
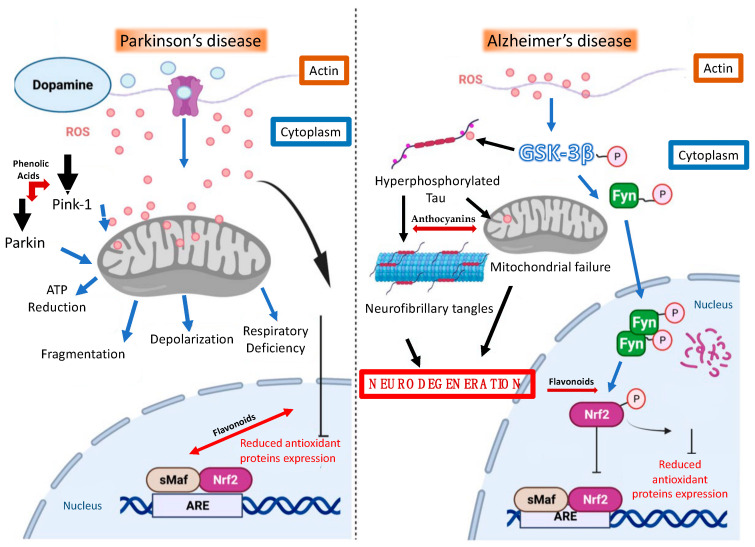
Mechanisms of Alzheimer’s disease and Parkinson’s disease and those of some components of the MD such as phenolic acids, anthocyanins, and flavonoids. Control over the Nrf2 pathway in neurons. Contribution of Nrf2 to the pathogenesis of Parkinson’s disease and Alzheimer’s disease. With Parkinson’s disease, an increase in dopamine release may have an impact on mitochondrial function, resulting in an increase in ROS levels that affect Nrf2 activity and the body’s response to oxidative damage. Moreover, the decline in Parkin and PINK expression levels discovered in PD may have an impact on the function of the mitochondrial system by causing depolarization, fragmentation, a respiratory deficit, and a reduction in ATP. These changes will have an impact on synaptic function and contribute to the cognitive decline and neurodegeneration seen in PD. In AD, the protein GSK-3, a Chinese medicine that promotes abnormal tau protein phosphorylation, promotes the degradation of Nrf2 as part of the proteasomal activity through the phosphorylation of Fyn. Moreover, in AD, activation of GSK-3 results in tau hyperphosphorylation, which may affect mitochondrial function. Following that, a buildup of tau pathological forms may lead to the development of neurofibrillary tangles (NFT), which are seen as a distinctive sign of AD.

The MD bioactive compounds act at various levels of both disease pathways, as shown in Table 3 and in Figure 2 [102].

## 6. Flavonoids

**Figure 3:**
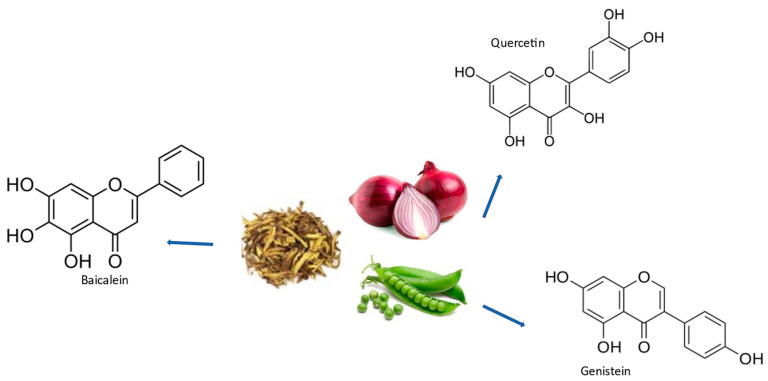
Chemical structures and some origins of baicalein (Scutellaria Baicalensis roots), quercetin (onions), and genistein (peas).

Flavonoids (some examples in Figure 3. Refs. [17,131,132]) are found in a wide variety of diets including vegetables, fruits, nuts, seeds, and beverages such as tea, coffee, and wine [133]. Flavonoid antioxidant activity can protect against free radical damage by scavenging ROS, activating antioxidant enzymes, inhibiting oxidases (such as xanthine oxidase [XO], cyclooxygenase [COX], lipoxygenase, and phosphoinositide 3-kinase [PI3K] [134]), and reducing α-tocopherol radicals [135]. Flavonoids are divided into six main subclasses (flavones, Flavonols, flavanones, flavanols, isoflavones, and anthocyanins). Studies have shown that flavonoids such as baicalein, quercetin, and rutin [136,137,138] can have several positive health effects, including a decreased risk of PD, despite their limited bioavailability. Their physiological actions, which include antioxidative, anti-inflammatory, anti-apoptotic, and lipid-lowering qualities, are the reason for this. The ability of flavonoids such as baicalein to reduce the elevated striatal baseline glutamatergic strength seen in PD by reducing presynaptic glutamate release and the reassembly of postsynaptic glutamate receptor subunits has been shown to be associated with their neuroprotective effects [103]. Another important flavonoid is quercetin, which exhibits senolytic activity. Senolytic flavonoids, such as quercetin, act in part by inhibiting members of the BCL-2 family, such as BCL-xL, as well as HIF-1α and other components of the senescent cell anti-apoptotic pathway (SCAP) network [139]. In the study by Zhang et al., it was proposed how substances with senolytic activity, such as quercetin, alleviate Aβ-associated oligodendrocyte progenitor cell senescence and cognitive deficits in an Alzheimer’s disease model [140]. Pro-inflammatory cytokine levels decreased as a result of the senolytic therapy, which may partly be attributable to the elimination of oligodendrocyte progenitor cells (OPCs) with the Senescence-Associated Secretory Phenotype (SASP) [140]. The potential beneficial effect of flavonoids in the brain appears to be linked to their ability to interact with glial signaling and intracellular neuronal pathways, triggering neuronal regeneration, increasing existing neuronal functions, protecting vulnerable neurons, or influencing the cerebrovascular and peripheral system [136,137,138,141]. Acetylcholine (ACh) is the most versatile neurotransmitter that plays its role in transmission of impulse across different neurotransmitters. Some studies have shown low levels of ACh in Alzheimer’s disease brains, and cholinesterase inhibitors not only boost ACh levels but also conduct impulse transmission at synaptic connections [142,143]. A lot of flavonoids have been employed as inhibitors of AChE, but among them, quercetin was found to be a potent inhibitor of AChE [144,145]. Another flavonoid, baicalein, exhibits high neuroprotective properties and greatly improves synaptic plasticity and memory impairments in an AD animal model [146,147].

## 7. Anthocyanins

**Figure 4:**
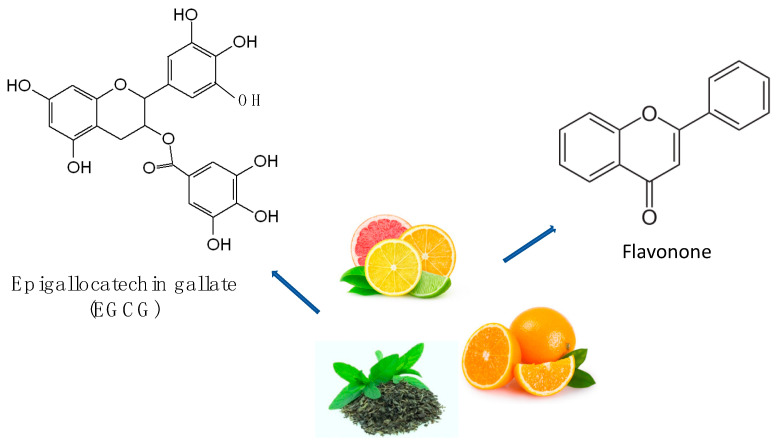
Chemical structures and some origins of epigallocatechin gallate [155] and flavonone [156], which are derived from green tea and citrus fruits, respectively.

**Figure 5:**
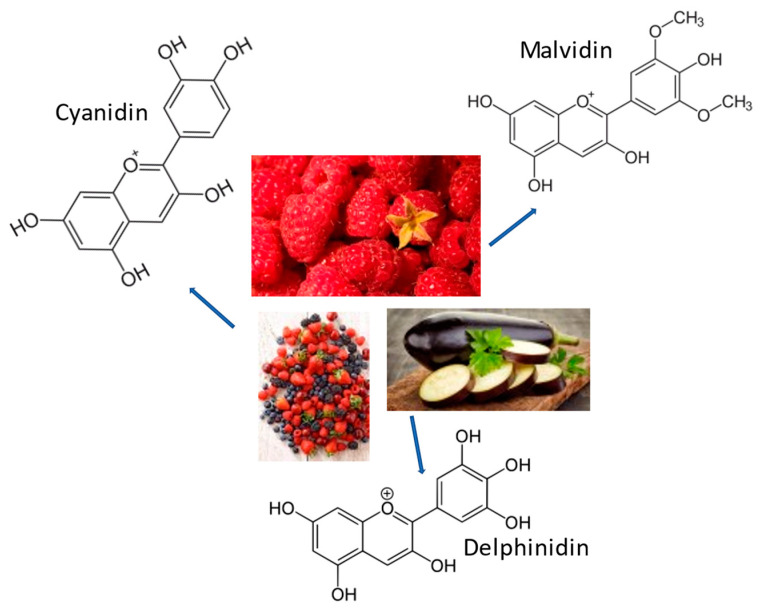
The chemical structures and some origins of cyanidin (red fruits), malvidin (strawberries), and delphinidin (eggplant). They express their activity in ionic form.

The most prevalent phenolic chemicals in nature are flavonoids (Figure 4) (anthocyanins, flavanols, flavonols, flavanones, flavones, chalcones, and dihydroxy chalcones) in polyphenols [148,149,150,151]. Anthocyanins (some examples in Figure 5 [149,150,151]) belong to the flavonoid group and are responsible for the red, violet, and blue color of many fruits and vegetables. Fruit residues, such as grape, mulberry, and raspberry, are rich in anthocyanins. Anthocyanins ameliorate oxidative stress by lowering free radical production and lipid peroxidation [152]. The complex mechanisms by which anthocyanins can directly scavenge free radicals, prevent the formation of reactive oxygen species (by forming chelating compounds with metals, they inhibit redox reactions and inhibit xanthine oxidase and NADPH oxidase), or encourage the release of antioxidant enzymes are what give them their antioxidant properties [153]. The antioxidant activities of phenolic substances are linked to biological effects such as anti-inflammatory and antibacterial actions. To quickly assess the strength of the possible antioxidant activity of the extracts of fruit samples from the investigated cultivars, antiradical and reductive activity in vitro experiments are used [154].

AD and other dementias are linked to cardiovascular and metabolic health risk factors, such as obesity and insulin resistance in middle age. Because anthocyanins are protective against CVD and T2DM risks, higher anthocyanin consumption may be related with a lower risk of Alzheimer’s disease in old age [157]. According to Seeram et al., the anthocyanin component in cranberry fruit extracts suppressed inflammatory processes by reducing prostaglandin synthesis by inhibiting COX [158]. Furthermore, anthocyanins increase the activation of the FKBP52 protein, which has an affinity for phosphorylated tau protein and prevents its aggregation [152]. Anthocyanins lower the intracellular Ca^2+^ ion concentration and inhibit caspase-3, which regulates neuronal apoptosis [159]. They also protected against memory loss as assessed by behavioral tests and measurement of anxiety, memory, and motor functions [51]. In addition, anthocyanins inhibited excessive synthesis of nitrogen reactive species in the cerebral cortex and hippocampus [160]. The neuroprotective effects of bioactive compounds present in berries were also suggested in experimental studies [161,162]. Extracts rich in anthocyanins have been shown to influence autophagy in recent research [163]. Toxic aggregates and misfolded proteins must be removed from the intracellular space by this process, in addition to proteasomal degradation, in order to prevent neuronal death. Both in vitro and in vivo studies have shown that acai berry extract is effective at promoting autophagy [164,165,166]. Recent research suggests that deficiencies in the autophagy–lysosome pathway, a key system for clearing misfolded proteins or damaged organelles, may precede the development of amyloid plaques or neurofibrillary tangles [167]. Additional research discovered that the autophagy regulatory protein Beclin1 and the lysosomal peptidase cathepsin D (CD), which are involved in the clearance of both Aβ peptides and tau protein, were diminished in AD brain tissues [168]. By causing damage to neurons and other biological components, oxidative stress and neuroinflammation cause neurodegeneration. Anthocyanins influence these metabolic pathways, enhancing antioxidant and anti-inflammatory defenses as well as maintaining normal hippocampus function, which lessens cognitive decline in mice given a high-fat diet (HFD), which was connected with elevated p-ULK1, Beclin1, and LC3-II and downregulated p62 [169]. Numerous studies have discovered that anthocyanins, a class of naturally occurring phenolic chemicals prevalent in berries, encourage autophagy [170]. Proto-catechuic acid (PCA) may have a major role in the bioactivity of anthocyanins, at least in part. ROS and caspase-3 levels in neural stem cells were shown to be reduced by PCA, while the increase in cytoplasmic Ca^2+^ concentration was observed to be inhibited [171].

## 8. Polyphenols

**Figure 6:**
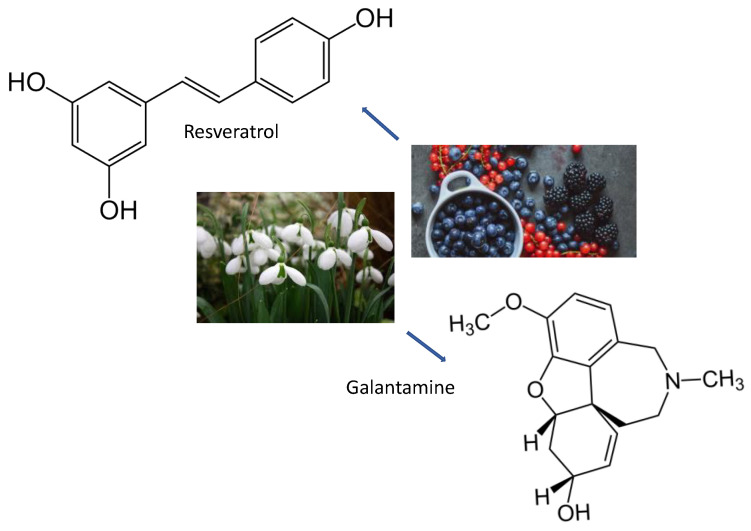
The chemical structures and some origins of galantamine, an alkaloid extracted from bulbs of species in the family Amaryllidaceae, and resveratrol, which is obtained from grapes.

The most prevalent antioxidants in the diet are polyphenols (some examples in Figure 6). The overall consumption of polyphenols from food might be as high as 1 g/d, which is significantly greater than the total dietary intake of all other groups of phytochemicals and recognized dietary antioxidants combined [172]. Polyphenols [173,174] (PPs) have recently been identified as the major plant-based bioactive molecules against a variety of diseases, including metabolic disorders, cardiovascular and neurological disorders, and some types of cancer [175,176,177,178,179,180,181,182]. PPs are naturally occurring complexes found in a variety of plants such as herbs, tea, fruits, and vegetables. These chemicals are important in plant pigmentation, growth, UV ray protection, and disease protection [183]. For its high metabolic activity, the brain is particularly susceptible to cellular damage caused by ROS. This is due to increased oxygen absorption [184] in order to satisfy higher energy needs and low quantities of antioxidant enzymes, making cells more sensitive to oxidation. The high levels of ROS in the brain are created by damaged mitochondria that produce less ATP and increase the ROS levels, affecting synaptic and non-synaptic communication between neurons and glia, leading to neuroinflammation and neuronal death, which leads to neurodegeneration [184,185]. Oxidative stress and damage to brain macromolecules are inherent processes in neurodegenerative diseases. The antioxidant properties of many polyphenols are purported to provide neuroprotection. The impacts of polyphenols on cognition and neurodegenerative processes appear to be mediated via interactions with neuronal and glial signaling pathways that influence gene expression and interfere with cell death mechanisms [186]. Polyphenols have been proven to have antioxidative properties, either directly by scavenging free radicals or indirectly by enhancing the capacity of the endogenous defense system. Among polyphenols, dihydrocaffeic acid has been discovered to have free radical scavenging properties [130]. The primary mechanism connecting and promoting the other pathogenic mechanisms of PD is the aggregation of α-Syn. Using the right inhibitors to maintain α-Syn proteostasis effectively prevents PD. In this work, two sets of xanthone–polyphenolic acid hybrids were created. The hybrids have stronger binding energies with α-Syn residues and a conjugated skeleton that resembles a sheet. The inhibitory action extends throughout the aggregation process, from lag phase to stationary phase, by stabilizing the conformation of α-Syn proteostasis and preventing β-sheet aggregation. Preliminary studies on the mechanism suggest that inhibitors could rapidly and randomly bind to the specific site closed to the β-sheet domain in the fibril, causing instability and collapse of the protein fibril, resulting in a complex system with aggregates of different sizes and monomers. [187]. Pathological α-Syn aggregation kinetics undergoes three steps, including the lag phase, the elongation phase, and the stationary phase, despite being altered by a variety of circumstances [188]. The lag phase, in which α-Syn monomers gradually assemble to form oligomer nuclei, is the rate-limiting process. Undoubtedly, any inhibitor that prevents the production of oligomer nuclei will be most effective during the lag period. On the other hand, inhibitors that can break down or disassemble α-Syn fibrils during the elongation phase or stasis phase may have beneficial medical consequences [189].

Recently, there has been a lot of attention placed on researching natural medicine as an AD treatment. For instance, the tertiary alkaloid galantamine, which has cholinesterase inhibitory qualities and the capacity to improve cholinergic function and alleviate memory problems, is now used in the treatment of AD [190]. Another polyphenol is represented by Rivastigmine; it is a semi-synthetic drug approved by the FDA as a cholinesterase inhibitor recommended for mild-to-moderate AD [191]. According to this study, dietary polyphenols may activate one or more pathways associated with adaptive cellular stress responses [192,193]. According to Calabrese et al., polyphenols in particular helped to increase the expression of genes that are good for you, such as Nrf2 genes [194]. It has been demonstrated that Nrf2 has a great affinity for the antioxidant response element (ARE) and participates in the upregulation of genes that regulate cellular redox levels and the cell’s defense system against oxidation [195]. Additionally, one of the most important polyphenols in this field is resveratrol. Resveratrol is a polyphenol that occurs naturally in red grapes, peanuts, and many other plant species. Administration of resveratrol to transgenic mouse models of AD reduces behavioral impairments and aging-related central nervous system (CNS) Aβ deposition [196,197]. Although the precise mechanisms are yet unknown, SIRT1 is crucial for the development and differentiation of neurons and guards against apoptosis by deacetylating and suppressing p53 activity [198]. Resveratrol decreases M1 microglia activation, which is involved in the start of neurodegeneration, and boosts Th2 responses by raising anti-inflammatory cytokines and SIRT1 expression. These actions together result in anti-inflammatory benefits [198].

### Phenolic Acids

**Figure 7:**
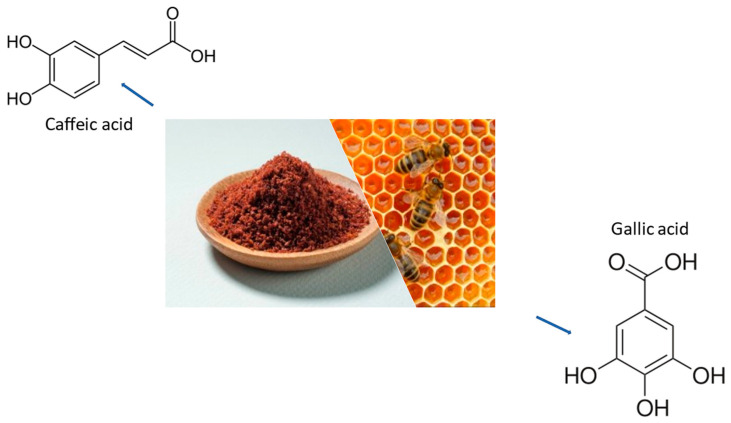
The chemical structures and some origins of some molecules that are part of phenolic acids. Caffeic acid is a derivative of cinnamic acid and is obtained from coffee while gallic acid is obtained from honey.

Phenolics [199,200] make up a sizable group of secondary metabolites in cereal grains (some examples in Figure 7). Insoluble phenolic acids would be accessible in the intestine after digestion by the enzymes (lipase, amylase, protease), and some would be released in the colon by the colonic microflora. Soluble phenolic acids would become absorbed in the stomach and small intestine for distribution to the whole body with concurrent health benefits such as inhibition of low-density lipoprotein, and cholesterol and liposome oxidation [201]. Two primary degradation processes that eliminate aberrant and unneeded proteins in order to preserve intracellular protein homeostasis are the autophagosome–lysosome route and the ubiquitin–proteasome system [202]. Autophagy represents a phylogenetically conserved eukaryotic degradative process which plays a crucial role in cellular homeostasis. These phytochemicals may also serve as autophagy activators, which might explain some of their favorable effects in Parkinson’s disease, such as inhibiting α-Syn aggregation. Despite the fact that α-Syn is a substrate of both autophagy and the proteasome, α-Syn clearance is carried out by autophagy when the proteasome is compromised, indicating that α-Syn may be a preferred substrate of autophagy [203]. For example, *Eucommia ulmoides Oliver* (EuO) leaf extracts (also known as EEuOL) may have anti-PD action. Inducing autophagy may have been the mechanism by which EEuOL reduced PD-like symptoms, and the phenolic acids in EEuOL may have contributed to this biological process, suggesting that EEuOL may be a promising therapeutic candidate for PD. The main EEuOL ingredients comprising chlorogenic acid, neochlorogenic acid, and cryptochlorogenic acid may help to activate autophagy; autophagy regulators Pink1 and Beclin1 showed interaction with most tested compounds. These compounds successfully entered the binding pockets of Pink1 and Beclin1 after molecular docking. This result suggests that autophagy is involved in the anti-PD activity that EEuOL exerts [168]. Secondary metabolites derived from medicinal plants showed potential against dementia and AD. Herbs such as *Scutellaria baicalensis*, *Ginkgo biloba*, *Hypericum perforatum*, *Curcuma longa*, *Lavandula angustifolia*, *Trigonella foenum-graecum*, and *Rosmarinus officinalis* have therapeutic potency, AChE inhibitory activity, antioxidant and anti-inflammatory capacity, and redox metal-chelating activities; they also inhibit Aβ aggregation and hyperphosphorylated tau proteins [204]. AD involves Aβ, which is the major component of senile plaques, produced due to the sequential action of β- and γ-secretases on the amyloid precursor protein (APP) through the amyloidogenic cleavage pathway. The plant-derived bioactive molecules (polyphenols, carotenoids, glucosinolates) were used for their antioxidant and anti-inflammatory effects to decrease protein aggregation, ameliorate cognitive ability, and regulate signaling pathways. Carito et al. explored the effect of the administration of bioactive molecules of the polyphenol class from olive leaves on male mouse neurotrophic proteins. In particular, the study suggests that nerve growth factor (NGF) and brain-derived neurotrophic factor (BDNF) and GDNF are involved in AD pathology. The administration of these polyphenols exhibited a key role in synaptic growth and protected neurons from damage [205]. Extra virgin olive oil includes 36 phenolic compounds, including tyrosol, hydroxytyrosols, oleocanthal, and oleuropein, as well as carotenes. These phenolic chemicals penetrate the brain and exert neuroprotective effects via antioxidant, anti-apoptotic, and anti-inflammatory mechanisms. According to extensive research, hydroxytyrosol functions as a scavenger of reactive free radicals, resulting in neuroprotective effects on brain cells during oxidative stress [206,207].

## 9. Conclusions

As the population ages, neurodegenerative diseases are more common. Neurodegenerative diseases are predominantly idiopathic disorders of unknown pathogenesis, such as AD and PD. There is currently no known cure for neurodegenerative diseases, while current medications may help some patients with their physical or mental symptoms. Inflammation, oxidative stress, and excess reactive oxygen play an important role in the progression of neurodegenerative disorders. Due to the brain’s high metabolic activity and reduced capacity for cell regeneration, oxidative stress is a crucial component in the onset of neurodegenerative diseases. Mitochondria that are not properly functional can kill cells in several ways, including interfering with energy metabolism and electron transport, accelerating the mitochondrial permeability transition, and releasing or activating proteins that induce apoptosis. All these pathways may help explain how mitochondrial dysfunctions lead to neuronal degeneration or dysfunction in degenerative diseases such as Parkinson’s disease and Alzheimer’s disease. Dietary interventions have been shown in the literature to regulate mitochondrial ROS production, detoxification, and repair of oxidative damage [208]. Because of their potential for neuroprotection, antioxidant and anti-inflammatory effects, and mitochondrial homeostasis to resist neuroinflammatory disorders associated with mitochondrial dysfunction, bioactive compounds have attracted the interest of scientists. In conclusion, these preliminary studies suggest that the Mediterranean diet is one of the greatest diet patterns for lowering the risk of neurological disorders [34,209,210]. Long-term consumption of plant foods, grains, legumes, fish, olive oil as the major source of fat, and a modest amount of red wine characterizes this dietary pattern. The molecular processes for chronic disease prevention by a Mediterranean pattern are due to the large levels of antioxidants, polyphenols, and other substances found in the Mediterranean diet, such as monounsaturated and polyunsaturated fatty acids. Current research suggests that more long-term, double-blind, randomized controlled trials on a large human population are required to promote the Mediterranean diet. This might help determine whether better adherence to this diet can help avoid or postpone the development of neurodegenerative diseases. With the development of more practical and comprehensive quality control guidelines to ensure the safety and efficacy of natural product therapies, as well as new approaches and strategies to facilitate access to the central nervous system of these neuroprotective agents, such as the incorporation of nanotechnology in the delivery of natural products, natural product therapy could play an essential role in the prevention and treatment of AD and PD and could promote the longevity and healthy aging of most people. 

## Figures and Tables

**Table 1:** Main foods that can be found in the Mediterranean diet and the bioactive compounds in which they are rich.

Foods	Sources	Reference
Legumes	Phenolic acids, anthocyanins/anthocyanidins, vitamin C	[36]
Milk and derivatives	Mineral salts, calcium, water-soluble and hyposoluble vitamins, fats and proteins	[37]
Vegetables	Vitamins (vitamin E, vitamin C), minerals (zinc and selenium), antioxidants, phytoestrogens, dietary fiber, flavonoids	[38]
Olive Oil	Hydrocarbons, phytosterols, fat-soluble vitamins, polyphenols	[39]
Fruits	sugar, fiber, vitamins and minerals, phenolic content	[40]
Meat	Potassium, calcium and iron, vitamin A, B vitamins, vitamin D, vitamin K, chromium, copper, folic acid, magnesium, selenium, n-3 fatty acids	[41]
Fish	Mineral salts (calcium, phosphorus, and iodine), vitamin A, B vitamins, vitamin D, proteins	[41]
Red Wine	It is a healthy source of antioxidants and its active ingredient, resveratrol. In addition, it is rich in vitamins and minerals.	[42]

**Table 2:** Main foods that can be found in the Mediterranean diet and their mechanisms in each disease.

Components of Mediterranean Diet	Effects on Cognitive Performance in Parkinson’s Disease	Effects on Cognitive Performance in Alzheimer’s Disease	Reference
Legumes	Antioxidants can help neutralize free radicals produced in the brain, consequently also reducing inflammation.	They can reduce oxidative stress and inflammation in the brain.	[43]
Red wine	It contains antioxidant and anti-inflammatory properties. Its constituent resveratrol has been associated with the reduction of oxidative stress, the inhibition of inflammation, and the promotion of neuroplasticity. It appears to affect beta-amyloid plaques.	Some compounds found in red wine, such as resveratrol and quercetin, may act as inhibitors of the enzyme monoamine oxidase B (MAO-B), which has been implicated in the degeneration of brain cells in Parkinson’s disease.	[44,45]
Vegetables	They can help reduce the buildup of beta-amyloid plaques in the brain. Plants are an important source of folic acid, which can help reduce levels of homocysteine, an amino acid associated with Alzheimer’s disease. They contain glucosinolate sulfates, which can promote the production of enzymes that eliminate toxic substances from the brain.	They contain vitamin C, vitamin E, and beta-carotene, which can protect brain cells from oxidative stress—one of the factors that can contribute to the degeneration of dopaminergic cells in the brain, responsible for the symptoms of Parkinson’s disease.	[46,47]
Olive Oil	It is rich in oleic acid, a monounsaturated fatty acid that may reduce the risk of cardiovascular and inflammatory diseases. It is rich in polyphenols, antioxidant compounds that can protect brain cells from oxidative damage. It appears to be able to reduce the formation of amyloid plaques in the brain.	It contains antioxidant compounds that may protect brain cells from oxidative stress and inflammation. Hidrox, a compound extracted from olives, can significantly ease Parkinson’s motor symptoms, reduce alpha synuclein buildup, and slow neurodegeneration in animal models of the disease.	[48,49]
Fruits	They contain antioxidants which help protect brain cells from oxidative damage. They also contain many anti-inflammatory compounds, including polyphenols. Chronic inflammation has been implicated in disease pathogenesis, and reducing inflammation may have protective effects on the brain. Certain compounds found in fruit, such as flavonoids, may help regulate the central nervous system and improve cognitive and motor function.	They can reduce the risk of amyloid-beta accumulation: Some studies have suggested that compounds found in fruit, such as polyphenols, may reduce the accumulation of amyloid-beta proteins in the brain.	[50,51]
Fish	It is rich in omega-3 fatty acids, especially eicosapentaenoic acid (EPA) and docosahexaenoic acid (DHA), which have been shown to have beneficial effects on brain health and disease prevention. Omega-3s can reduce chronic inflammation in the brain, improve cognitive function, including memory, protect brain cells from damage, and reduce the accumulation of abnormal proteins in the brain, such as tau and amyloid-beta proteins.	It contains vitamin D, which can influence the function of dopamine receptors in the brain and can have protective effects on brain cells. Omega-3 fatty acids can reduce inflammation in the brain and protect brain cells from oxidative damage.	[32,52,53,54]

**Table 3:** Some bioactive compounds and the pathways involved in their mechanism of action.

Substances	Natural Source	Pathways Involved	References
Apigenin	Parsley	Nrf2	[103]
Baicalein	Roots Of Scutellaria Baicalensis	Nf-Kb/DJ-1	[103,104,105]
Luteolin	Parsley, Radicchio, Celery	Nrf2	[103,106,107,108,109,110,111,112,113,114,115]
Quercetin	Many Fruits, Vegetables, Leaves, Seeds, And Grains; Capers, Red Onions	MAPK/AKT/PI3K ERK/CREB	[103,116,117]
Isoquercetin	Mangifera Indica	MAPK/AKT	[103]
Rutin	Citrus	PI3K/Erβ	[103,118]
Kaempferol	Kale, Beans, Tea, Spinach, And Broccoli	DJ-1	[103]
Naringin	Citrus Fruits, Especially In Grapefruit	Nrf2/ARE	[103]
Naringenin	Grapefruit	Nrf2/ARE	[103]
Hesperidin	Lemons And Sweet Oranges	GSH/Bcl-2	[103]
Epigallocatechin-3-Gallate	Green Tea	PKC/MAPK/PI3K Akt MEK/ERK1/2	[119]
Daidzein	Legumes, Especially In Soybeans	Bcl-2	[103]
Genistein	Many Other Vegetables, Fruits, Nuts, Peas, Lentils, And Seeds	Bcl-2	[103]
Quercetin	Parsley, Citrus	MAPK/AKT/PI3K ERK/CREB	[120]
Flavanone	Orange	Phosphatidylinositol 3-Kinase/Akt	[121]
Cyanidin	Red Berries Including Grapes, Bilberry, Blackberry	JNK/BDNF	[122]
Galantamine	Bulbs Of Galanthus Nivalis	Cholinesterase Inhibitor	[123]
Resveratrol	Skin Of Grapes, Blueberries, Raspberries, Mulberries, And Peanuts	SIRT1/PGC-1/PI3K/Akt	[117,123,124,125,126,127,128,129]
Caffeic Acid	Honeybee Hives	Counteracted Aggregation	[130]
Chlorogenic Acid	Coffee And Black Tea	Protected Against NO Effects	[130]
Gallic Acid	Gallnuts, Sumac, Witch Hazel, Tea Leaves, Oak Bark	CAT/GSH/SOD	[130]

## Data Availability

Not applicable.
